# The retinal phenotype in primary hyperoxaluria type 2 and 3

**DOI:** 10.1007/s00467-022-05765-1

**Published:** 2022-10-19

**Authors:** Johannes Birtel, Roselie M. Diederen, Philipp Herrmann, Sophie Kaspar, Bodo B. Beck, Sander F. Garrelfs, Bernd Hoppe, Peter Charbel Issa

**Affiliations:** 1 Oxford Eye Hospital, Oxford University Hospitals NHS Foundation Trust, Oxford, OX3 9DU UK; 2 Nuffield Laboratory of Ophthalmology, Nuffield Department of Clinical Neurosciences, University of Oxford, Oxford, UK; 3 Department of Ophthalmology, University Medical Center Hamburg-Eppendorf, Hamburg, Germany; 4 Department of Ophthalmology, University Hospital of Bonn, Bonn, Germany; 5 Department of Ophthalmology, Amsterdam University Medical Centers, University of Amsterdam, Amsterdam, the Netherlands; 6 Institute of Human Genetics, Center for Molecular Medicine Cologne, and Center for Rare and Hereditary Kidney Disease, University Hospital of Cologne, University of Cologne, Cologne, Germany; 7 Emma Children’s Hospital, Amsterdam UMC, Department of Pediatric Nephrology, University of Amsterdam, Amsterdam, the Netherlands; 8 German Hyperoxaluria Center Bonn, Kindernierenzentrum Bonn, Bonn, Germany

**Keywords:** Primary hyperoxaluria, Kidney failure, Plasma oxalate, Retina, Phenotyping, Optical coherence tomography

## Abstract

**Background:**

The primary hyperoxalurias (PH1-3) are rare inherited disorders of the glyoxylate metabolism characterized by endogenous overproduction of oxalate. As oxalate cannot be metabolized by humans, oxalate deposits may affect various organs, primarily the kidneys, bones, heart, and eyes. Vision loss induced by severe retinal deposits is commonly seen in infantile PH1; less frequently and milder retinal alterations are found in non-infantile PH1. Retinal disease has not systematically been investigated in patients with PH2 and PH3.

**Methods:**

A comprehensive ophthalmic examination was performed in 19 genetically confirmed PH2 (*n* = 7) and PH3 (*n* = 12) patients (median age 11 years, range 3–59).

**Results:**

Median best corrected visual acuity was 20/20. In 18 patients, no retinal oxalate deposits were found. A 30-year-old male with PH2 on maintenance hemodialysis with plasma oxalate (Pox) elevation (> 100 µmol/l; normal < 7.4) demonstrated bilateral drusen-like, hyperreflective deposits which were interpreted as crystallized oxalate. Two siblings of consanguineous parents with PH2 presented with retinal degeneration and vision loss; exome-wide analysis identified a second monogenic disease, *NR2E3*-associated retinal dystrophy.

**Conclusions:**

Retinal disease manifestation in PH2 and PH3 is rare but mild changes can occur at least in PH2-associated kidney failure. Decline in kidney function associated with elevated plasma oxalate levels could increase the risk of systemic oxalosis. Deep phenotyping combined with genomic profiling is vital to differentiate extrarenal disease in multisystem disorders such as PH from independent inherited (retinal) disease.

**Graphical abstract:**

**Figure:**
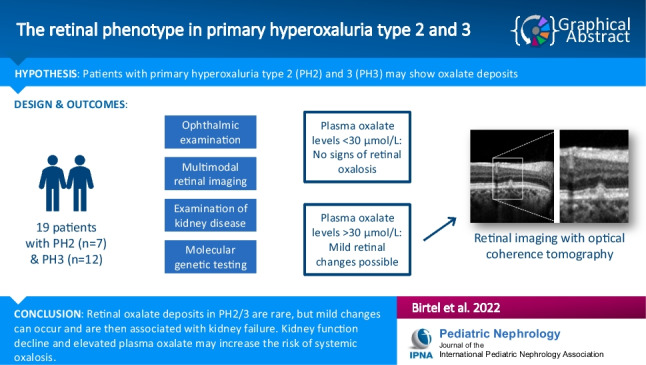
A higher resolution version of the Graphical abstract is available as Supplementary information

**Supplementary Information:**

The online version contains supplementary material available at 10.1007/s00467-022-05765-1.

The primary hyperoxalurias (PH) are three rare inherited disorders of glyoxylate metabolism characterized by endogenous overproduction of oxalate [1, 2]. As oxalate cannot be metabolized by humans, it is excreted mostly by the kidneys and induces recurrent kidney stone formation, progressive nephrocalcinosis, or both. This leads to chronic kidney disease (CKD), and eventually kidney failure [1, 2]. Beyond the kidneys, oxalate crystal deposition may also affect various organs, most notably the skeleton, heart, and the eyes [1, 2].

Depending on the molecular disease cause, three subtypes of PH can be differentiated [2]. The most common and most severe form is PH1; its heterogeneous clinical presentation includes infantile PH with kidney failure in the first months of life with extensive systemic oxalosis, as well as non-infantile PH with kidney failure onset during adulthood, sometimes leading to (severe) systemic oxalosis (bone, heart), but to a mild or absent retinal phenotype [2–4]. PH2 has a more favorable prognosis with later-onset kidney failure and milder systemic oxalosis, comparable to that of non-infantile PH1 patients [5]. In PH3, described as least severe, recurrent kidney stones in adulthood, impaired kidney function and even kidney failure were recently reported [6, 7].

Vision loss already at a young age and severe subretinal oxalate deposition is seen in all patients with infantile PH1, whereas patients with non-infantile PH1 usually present with no or milder retinal alterations (subretinal deposits) that usually do not affect visual function [3, 4, 8, 9]. If patients with PH2 and PH3 also develop retinal disease has not been investigated in detail. Characterization of extrarenal manifestation may be important in light of novel therapeutic options such as small interfering RNA, which might become available for PH2/PH3 as it is already the case for PH1 [10–13]. This study aims to investigate the ocular phenotype in patients with PH2 and PH3 and to determine its relation to kidney disease.

## Methods

This retrospective, cross-sectional multicenter study followed the tenets of the Declaration of Helsinki; informed written consent was obtained from each patient. Genetic testing confirmed the clinical diagnosis of PH2 or PH3 in all patients (Table 1).

**Table 1:** Summary of demographic, ophthalmic, kidney disease, and genetic findings

ID (#)	Diagnosis	Gender	Age at ocular examination (years)	BCVA		Refraction		Kidney disease						Genetic analysis		
				OD	OS	OD	OS	Age of first symptoms (years)	Age of PH diagnosis (years)	Plasma oxalate level (µmol/L)	eGFR (ml/min)	CKD (stage) at ocular examination	Transplantation history	Gene	Variant 1	Variant 2
Primary hyperoxaluria type 2																
#1	PH 2	f	4	20/25	20/25	+ 0.50/-0.25/19°	+ 0.50/-0.00/0°	1	1,5	7.5	128	-	-	*GRHPR*	c.103delG	c.103delG
#2	PH 2	m	8	20/20	20/20	+ 0.50/ + 0.25/114°	+ 0.50/ + 0.50/91°	1	1	5.3	111	-	-		c.103delG	958G > T
#3*	PH 2	f	11	20/40	20/40	nd	nd	1	2	5.5	105	-	-		c.287G > A	c.287G > A
#4*	PH 2	f	11	20/40	20/40	nd	nd	3	3	5.6	101	-	-		c.287G > A	c.287G > A
#5	PH 2	f	24	20/20	20/20	nd	nd	0.8	0.8	4.7	78	-	-		p.103delG	p.103delG
#6	PH 2	m	30	20/25	20/20	+ 0.25/-0.25/103°	+ 0.25/-0.50/80°	8	9	116.3	kidney failure	5	LKTx		c.344C > A	c.344C > A
#7	PH 2	m	59	20/25	20/20	-1.75/-1.00/93°	-2.25	21	57	54.5	12	5	2 × KTx		c.103delG	c.103delG
Primary hyperoxaluria type 3																
#8	PH 3	m	3	20/16	20/16	nd	nd	2	3	7.6	78	2	-	*HOGA1*	c.700 + 5G > T	c.700 + 5G > T
#9	PH 3	f	8	20/20	20/20	+ 0.25/-0.50/176°	+ 0.75/-0.50/3°	2	2	4.5	82	2	-		c.700 + 5G > T	c.634A > C c.661G > C
#10	PH 3	f	7	20/25	20/25	+ 0.75/-0.50/3°	+ 0.75/-0.50/168°	asymptomatic	1	5.6	73	2	-		c.700 + 5G > T	c.634A > C c.661G > C
#11	PH 3	m	9	20/50	20/25	+ 0.50/-2.50/176°	0/-2.00/6°	3	8	18.3	129	-	-		c.266G > A	c.266G > A
#12	PH 3	m	10	20/20	20/20	+ 0.50/-0.25/168°	+ 0.75/-0.25/176°	1	2	5.8	129	-	-		c.221 T > G	c.700 + 5G > T
#13	PH 3	m	11	20/20	20/20	+ 0.75/-0.50/7°	+ 1.25/-1.75/178°	1	1	3.4	117	-	-		c.728C > A	c.728C > A
#14*	PH 3	m	11	20/20	20/20	-0.75/-1.25/123°	+ 0.75/-0.75/180°	3	5	8.0	99	1	-		c.700 + 5G > T	c.944_946delAGG
#15*	PH 3	f	11	20/20	20/20	-1.00/-0.50/107°	-1.25/-0.25/82°	asymptomatic	5	9.5	92	1	-		c.700 + 5G > T	c.944_946delAGG
#16*	PH 3	f	13	20/20	20/20	-0.25/-0.25/105°	-0.25/-0.5/176°	asymptomatic	7	9.7	64	2–3	-		c.700 + 5G > T	c.944_946delAGG
#17	PH 3	m	29	20/25	20/20	+ 1.25/-1.00/1°	+ 1.50/-1.50/2°	15	24	22	104	1	-		c728C > A	c728C > A
#18	PH 3	m	39	20/20	20/20	-1.25	-1.0	28	33	8.2	43	3B	-		c.908G > A	c.908G > A
#19	PH 3	f	48	20/16	20/16	plano	-0.25/-0.25/178°	3	42	6.9	49	3A	-		c.700 + 5G > T	c.700 + 5G > T

*PH*, primary hyperoxaluria; *m*, male; *f*, female; *, siblings; *BCVA*, best corrected visual acuity; *OD*, right eye; *OS*, left eye; *PLT*, preferential looking test; *eGFR*, estimated glomerular filtration rate; *CKD*, chronic kidney disease; *LKTx*, liver-kidney transplantation; *KTx*, kidney transplantation; *nd*, not documented; *GRHPR*, glyoxylate and hydroxypyruvate reductase; *HOGA1*, 4‐hydroxy‐2‐oxoglutarate aldolase

From each patient, a history regarding visual symptoms and ocular conditions was obtained. Ophthalmic examination included best-corrected visual acuity (BCVA) testing, slit-lamp examination and indirect ophthalmoscopy after pupil dilation. Retinal imaging was performed using fundus photography, spectral-domain optical coherence tomography (OCT) imaging (Spectralis HRA-OCT Heidelberg Engineering, Heidelberg, Germany), and in selected cases fundus autofluorescence (AF) imaging [14, 15].

Comprehensive evaluation of the kidney disease at the time of retinal examination included analysis of the estimated glomerular filtration rate (eGFR) and plasma oxalate (Pox) levels. eGFR was calculated using the Bedside IDMS-traceable Schwartz eGFR equation in pediatric patients (< 18 years of age), and the CKD-EPD equation in adults (≥ 18 years of age) [16, 17].

## Results

Nineteen patients (9 female; 47%) from 15 families were included in this study, of whom 7 (37%) were diagnosed with PH2 and 12 (63%) with PH3. Median age at first symptoms was 1 year (interquartile range [IQR] 1–8 years) and median age of diagnosis was 3 years (IQR, 1.5–9 years) for patients with PH2, and 3 years (IQR, 1.5–9 years) and 5 years (IQR, 2–20 years) for PH3 patients, respectively. Three patients (#10, #15, #16) were asymptomatic, but were found to be affected after their younger sibling (#9, #14) was diagnosed with PH3.

At the time of ophthalmic examination, chronic kidney disease (CKD ≥ stage 2) was present in the 8 patients, with the 4 oldest patients (30, 39, 48, and 59 years of age) presenting with CKD 3A or worse (Table 1). The 30-year-old (#6) and 59-year-old patient (#7) with PH 2 were on maintenance hemodialysis (HD), patient #6 already for 17 months. Later, at age 32, he underwent combined liver-kidney transplantation which was performed based on severe systemic oxalosis. Patient #7 underwent two subsequent kidney transplantations prior to establishing diagnosis of PH2 and returned to HD one month before ophthalmic examination.

Median plasma oxalate levels were 5.6 µmol/L (IQR, 5.3–55 µmol/L) in patients with PH2 and 7.8 µmol/L (IQR, 5.7–9.7 µmol/L) in patients with PH3. The two patients with kidney failure (#6, #7) had markedly elevated plasma oxalate (#6: 116.3 µmol/L; #7: 54.5 µmol/L), whereas all others had plasma oxalate levels below 30 µmol/L, which is regarded as the threshold for plasma calcium-oxalate saturation [18]. Patient characteristics are summarized in Table 1.

### Retinal phenotype

Median age at ophthalmic examination was 11 years (IQR, 8–30 years). Seventeen patients reported no visual problems apart from mild refractive errors, and mean BCVA was 20/20 (range, 15/20–30/20). In two siblings (#3, #4), vision was bilaterally reduced to 20/40 (additional details below).

**Fig. 1:**
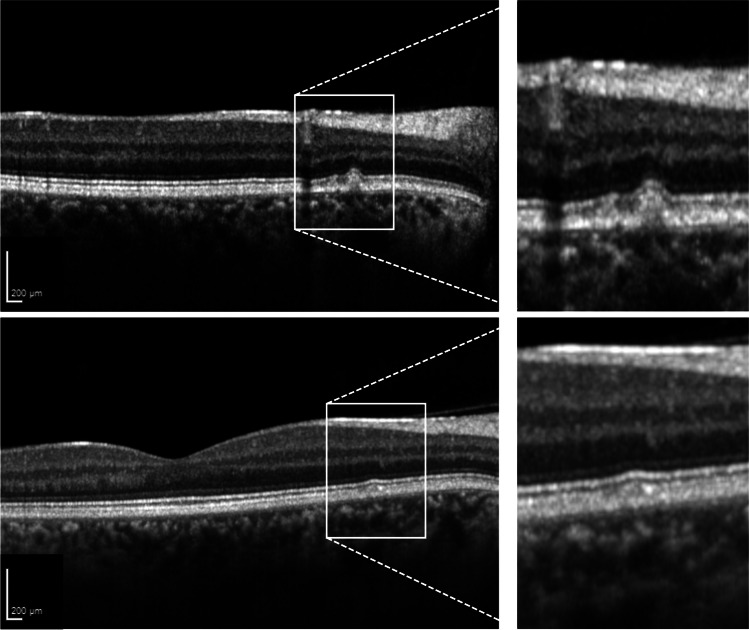
Retinal alterations identified in a patient (#6) with primary hyperoxaluria type 2 using optical coherence tomography (OCT) imaging. OCT imaging shows small focal hyperreflective subretinal lesions which were interpreted as crystallized oxalate. The overlying neurosensory retina is preserved

An unremarkable retinal phenotype without retinal oxalate deposits on multimodal retinal imaging was found in 16 patients (#1–2, #5, #7–19). In contrast, the 30-year-old patient with PH2 (#6) had drusen-like deposits in both eyes, primarily distributed around the optic disc (within a radius of two optic disc diameters), that were interpreted as crystallized oxalate. On OCT imaging, these deposits appeared as focal hyperreflective subretinal lesions (Fig. 1).

**Fig. 2:**
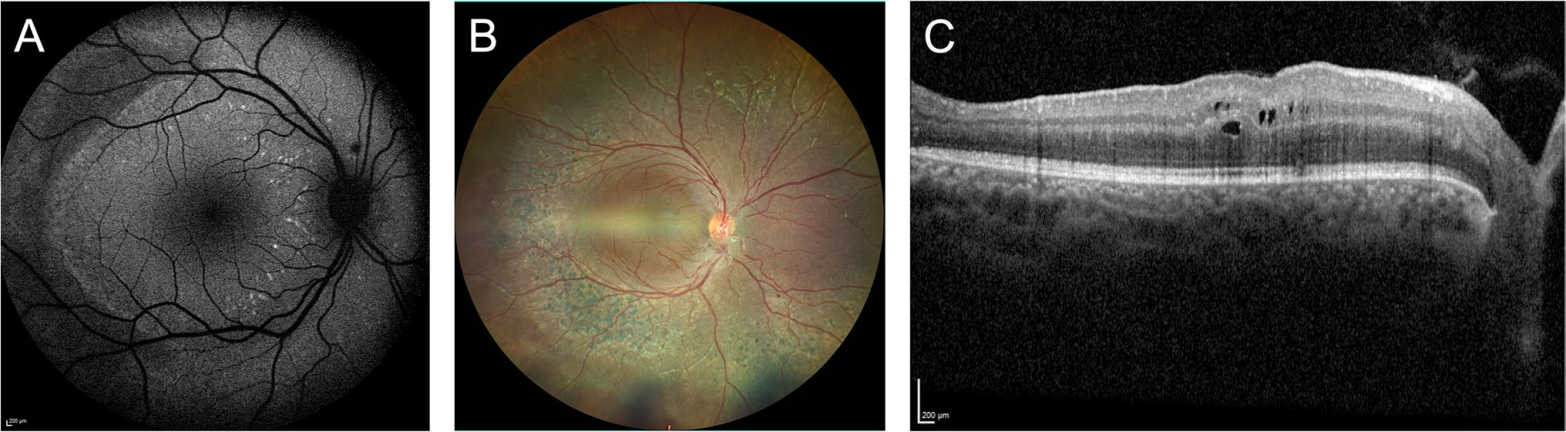
A retinal dystrophy associated with a homozygous splice-site mutation (c.119-2A > C) in the *NR2E3 *gene was present in patients #3 and #4. (**A**) Fundus autofluorescence of #3; (**B**) fundus photography; and (**C**) spectral-domain optical coherence tomography of #4

The retinal phenotype of two 11-year-old twins (#3, #4) with consanguineous parents differed from the other 17 patients (Fig. 2). They reported deterioration of visual function since early childhood and dilated fundus examination showed bilateral slightly attenuated blood vessels and mid-peripheral, nummular hyperpigmentations. AF imaging revealed reduced autofluorescence in the temporal (mid-) periphery and spots of increased autofluorescence mainly central to the proximal temporal arcades. OCT imaging showed abnormal inner retinal thickening, foremost in the nasal retina, cystoid macular changes, and small hyperreflective dots in the inner and outer retinal layers without shadowing. No deposits indicative of crystallized oxalate were seen. Overall, the retinal phenotype was more pronounced in patient #4 compared to #3. To assess a potential involvement of a second gene contributing to these severe retinal alterations, whole exome sequencing was performed and identified a homozygous acceptor splice mutation (c.119-2A > C) in the nuclear receptor subfamily 2, group E, member 3 (*NR2E3)* gene, which was previously reported in patients with enhanced S-cone syndrome [19].

## Discussion

In this group of 19 patients with PH2 and PH3, children and adolescents with low plasma oxalate levels and good kidney function exhibited no signs of ocular oxalosis. Out of two adult patients with PH2 and plasma oxalate levels above the presumed threshold for plasma supersaturation of 30 µmol/L [18], one was found to have mild subretinal deposits that were interpreted as oxalate deposits. Importantly, these deposits did not affect visual function. In PH3 patients who all had low plasma oxalate levels, no retinal alterations were found.

Hence, the retina in patients with PH2 and PH3 and plasma oxalate levels < 30 µmol/L usually shows no changes related to systemic oxalosis. In patients with plasma oxalate levels > 30 µmol/L, a mild phenotype may be present with deposits similar to those seen in patients with non-infantile PH1 [4]. This phenotype clearly differs from patients with infantile PH1 who develop severe oxalate deposits and subretinal fibrosis, often leading to vision loss already at a young age [3, 4, 8].

As kidney function rarely declines to kidney failure before the age of 15 years in PH2, absence of oxalate deposits may have been expected in the reported 4- and 8-year-old PH2 patients with good kidney function [5]. Later decline in kidney function associated with increased plasma oxalate levels could increase the risk of systemic oxalosis with subretinal oxalate deposition as described in PH1. At this mild end of the phenotypic spectrum, additional genetic and/or environmental modifiers might result in variable susceptibility. This could explain the retinal deposits in the 30-year-old PH2 patient (#6) who was on HD for 3 years before he underwent combined liver-kidney transplantation, whereas a 59-year-old (#7) patient with lower plasma oxalate levels, and a shorter period in kidney failure, did not show deposits. Further studies, that investigate systemic deposits, e.g., in the bones or heart, are vital and currently performed in the German Hyperoxaluria Center Bonn.

Subretinal oxalate deposits in patients with non-infantile PH1, PH2, and PH3 might also be residues from previous periods of high plasma oxalate levels, e.g., before liver-kidney transplantation or other means of treatment. Future longitudinal phenotyping with multimodal retinal imaging in (larger) patient cohorts would be required to gain further insights into whether subretinal deposits are indeed associated with a higher current or historical systemic and kidney disease burden. Moreover, the prognostic significance of the presence and severity of subretinal oxalate deposits, as well as their reversibility under therapy, will need to be investigated in future longitudinal studies.

Two siblings from a family with known consanguinity (#3, #4) presented with a history of vision problems and a retinal phenotype compatible with *NR2E3*-related retinal dystrophy, which is unrelated to systemic oxalosis. The twins carried a homozygous splice-site variant (c.119-2A > C) in intron 1 of *NR2E3* that has been tested in functional splicing assays and is predicted to lead to a skipping of exon 2 and the generation of a premature stop codon in exon 3 [20].

An extended phenotypic spectrum or co-occurrence of a second (monogenic) disease need to be considered in patients with findings that are not consistently identified in well-defined patients cohorts [21, 22]. Here, an independent molecular defect in *NR2E3* explained the retinal phenotype in 2 patients with PH. Exact phenotyping and molecular genetic analysis are required to correctly differentiate potential ocular manifestation of multisystem disorders such as PH from independent inherited (retinal) disease and for comprehensive genetic counselling.

Limitations of this study include its retrospective, cross-sectional study design, the overall young age of the included patients, the mostly good kidney function, and plasma oxalate levels mainly below the presumed threshold for plasma supersaturation. Therefore, additional and longitudinal studies are important, which may focus on patients with advanced disease. Such studies may also reveal factors influencing the retinal changes and whether or not retinal oxalate depositions are reversible.

## Supplementary Information

Below is the link to the electronic supplementary material.

Graphical Abstract (PPTX 866 KB)
