# Supplementary material for: The retinal phenotype in primary hyperoxaluria type 2 and 3
Source: Pediatr Nephrol. 2022 Oct 19;38(5):1485–90. doi: 10.1007/s00467-022-05765-1 (PMC10060347; doi:10.1007/s00467-022-05765-1)
Supplement: Supplementary file 1 — Graphical Abstract (PPTX 866 KB) [file 467_2022_5765_MOESM1_ESM.pptx]

## Slide 1
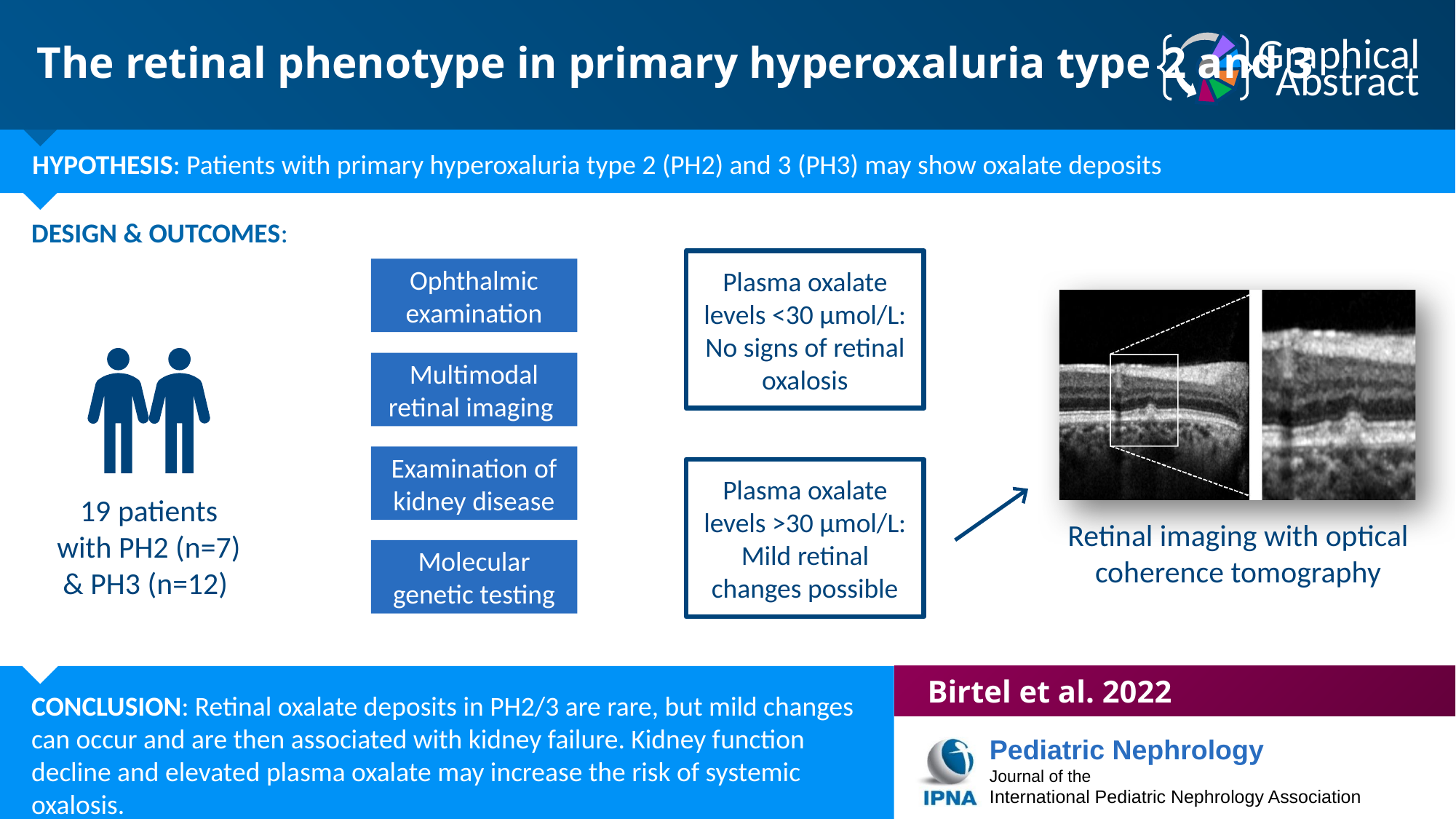

The retinal phenotype in primary hyperoxaluria type 2 and 3
HYPOTHESIS: Patients with primary hyperoxaluria type 2 (PH2) and 3 (PH3) may show oxalate deposits
DESIGN & OUTCOMES:
Plasma oxalate levels <30 µmol/L: No signs of retinal oxalosis
Ophthalmic examination
Multimodal retinal imaging
Examination of kidney disease
Plasma oxalate levels >30 µmol/L: Mild retinal changes possible
19 patients with PH2 (n=7) & PH3 (n=12)
Retinal imaging with optical coherence tomography
Molecular genetic testing
Birtel et al. 2022
CONCLUSION: Retinal oxalate deposits in PH2/3 are rare, but mild changes can occur and are then associated with kidney failure. Kidney function decline and elevated plasma oxalate may increase the risk of systemic oxalosis.
